# Pathways From Cyber Victimization to Adolescent Depression: A Latent Profile and Bridge Network Analysis

**DOI:** 10.1155/da/3544219

**Published:** 2026-09-26

**Authors:** Ergün Kara, Halil Aslan

**Affiliations:** ^1^ School of Medicine, Medical Sciences and Nutrition, University of Aberdeen, Aberdeen, UK, abdn.ac.uk; ^2^ Department of Educational Sciences, Siirt University, Siirt, Türkiye, siirt.edu.tr

**Keywords:** adolescent depression, bridge analysis, cyber victimization, latent profile analysis, network psychometrics, self-esteem, social media use

## Abstract

Cyber victimization is among the most consistent predictors of depression (DEP) in adolescence, yet research has largely treated both constructs as unidimensional totals, obscuring which specific victimization experiences activate which depressive symptoms and for whom. This study applied a person‐centered and network psychometric approach to map these pathways in a cross‐sectional community sample of 622 Turkish adolescents (55.0% female; *M*_aɡe_ = 13.90 years). Latent profile analysis (LPA) identified three distinct subgroups based on self‐esteem (SE), family affluence, and social media (SM) use: a low SE/moderate SM group (*n* = 395, 63.5%), a high SE/high DEP group (*n* = 115, 18.5%), and a high SM use group (*n* = 112, 18.0%). Depressive symptom networks differed significantly in global strength across profiles; the high SM use group showed the densest intersymptom connections. A bipartite bridge network analysis revealed that self‐harm/suicidal ideation was the strongest cross‐community bridge node (bridge expected influence = 0.143), despite being the most peripheral symptom within the DEP network itself. This dissociation suggests that the symptom is linked mainly to cyber victimization rather than to other depressive symptoms. Embarrassing messages was the strongest victimization‐side bridge node (EI = 0.095). These findings challenge the assumption that intranetwork centrality predicts intervention priority: Peripheral symptoms may function as critical entry points when external stressors drive activation.

## 1. Introduction

Depression (DEP) is one of the most prevalent and impairing mental health conditions during adolescence, affecting an estimated 13% of young people globally and representing a leading cause of disability in this age group [1]. Clinically, adolescent DEP is not a transient mood disturbance: It commonly presents with irritability alongside low mood and anhedonia, frequently co‐occurs with anxiety and behavioral difficulties, is closely linked to suicidal ideation and self‐harm, and carries a high risk of recurrence and of persistent functional impairment in education and social relationships that extends into adulthood [2]. Epidemiological evidence also indicates that a considerable proportion of adolescents who meet diagnostic criteria for major DEP do not receive DEP‐specific treatment, which makes the early identification of specific, modifiable risk pathways a clinical as well as a theoretical priority [3]. The developmental period from early to late adolescence is characterized by heightened social sensitivity [4], identity formation, and reliance on peer relationships. These conditions make young people particularly vulnerable to the psychological consequences of online harm. Adolescence is further characterized by a normative rise in risk‐taking and sensation‐seeking behavior, which increases from preadolescence, peaks in late adolescence, and declines thereafter, while self‐regulatory capacity continues to mature into the twenties [5]. This developmental imbalance shapes both how intensively young people engage with online environments and how much exposure to online harm they accumulate. Cyber victimization, defined as deliberate and repeated exposure to harmful online behaviors such as harassment, impersonation, and public humiliation, has emerged as one of the most consistent environmental predictors of adolescent DEP in cross‐sectional and longitudinal research alike [6, 7]. Meta‐analytic evidence reports consistent positive associations between cyber victimization and internalizing problems, including DEP. Pooled estimates across 55 reports and 257,678 adolescents ranged from *r* = 0.14 to 0.34 [8], and cybervictims showed almost threefold higher odds of DEP than nonvictims (OR = 2.73, 95% CI [2.25, 3.31]; [9]). What is less clear is the specificity of this relationship. The majority of existing studies regress a cyber victimization total score onto a DEP total score. This approach aggregates across qualitatively distinct forms of online harm and across symptom dimensions that may respond very differently to those harms [10–12]. Public humiliation differs from account hacking in its mechanisms of psychological injury; anhedonia likely responds to social rejection in ways that fatigue does not [13]. A methodology capable of resolving these within‐construct distinctions is therefore needed, both to advance theory and to identify which specific behaviors and symptoms constitute the most promising targets for prevention.

### 1.1. The Role of Self‐Esteem (SE) and Social Media (SM) Use

Two contextual factors consistently moderate the victimization‐DEP pathway: SE and SM use. SE refers to an individual’s subjective evaluation of their own worth [14]. Self‐related resources shape how far cyber victimization translates into psychological harm. Self‐efficacy and parental communication attenuate depressive symptoms following cyber victimization [15], and among Turkish gifted adolescents, SE and social support jointly mediate the association between cyber victimization and subjective well‐being [16]. Low SE plausibly intensifies the appraisal of negative online experiences as self‐relevant and threatening, and network evidence links cyber victimization specifically to social phobia and low SE [17]. Similarly, a recent meta‐analysis of 81 studies comprising 110,095 adolescents found that high levels of cyber victimization were associated with low SE [18]. The shame and worthlessness that characterize low SE map onto specific depressive symptom clusters, particularly the worthlessness and hopelessness dimension, rather than uniformly elevating all depressive symptoms [19, 20]. This observation raises a question that aggregate analyses cannot answer: Does low SE specifically strengthen certain symptom connections within the DEP network, making that network harder to exit once activated?

SM use adds a further dimension. High‐volume and problematic SM engagement travels closely with DEP, and the association runs in both directions. In a three‐wave study of Chinese adolescents, those with probable DEP at baseline were far more likely to show problematic SM use 2 years later (12.6% vs. 1.3%), and this difference operated through lower peer acceptance and the loneliness that followed from it [21]. In a cross‐national survey covering 42 countries, intense use and, more consistently, problematic use of SM were associated with an elevated risk of cyber victimization, although the strength of the association varied considerably between countries [22]. Among adolescent girls, Göksu et al. [23] found higher SM use and higher cyber victimization scores in those with major depressive disorder than in matched controls, with cyber victimization related to using SM to meet social‐competence needs and to lower perceived social support. Adolescents who use SM the most are also those most frequently victimized. Yet the structural consequences of this co‐occurring risk pattern for the organization of depressive symptoms have not been examined. Whether high SM use produces a qualitatively different DEP architecture, beyond simply increasing overall symptom severity, is an open empirical question.

### 1.2. A Network Psychometric Perspective

Network theory reconceptualizes mental disorders not as latent entities expressed through symptoms but as dynamic systems in which symptoms directly activate each other through feedback loops [24]. In a network representation, symptoms are nodes, and statistical associations between them are edges; a symptom with high centrality maintains many strong connections to other symptoms and therefore sustains the disorder state by continuously reactivating neighboring nodes. The clinical implications are meaningful. High‐centrality symptoms are theoretically more important intervention targets, since disrupting them propagates across the network. Low‐centrality symptoms can be disrupted without substantially altering the overall system.

Bridge centrality extends this logic to co‐occurring or comorbid conditions. Bridge nodes are symptoms that link one symptom community to another, functioning as entry points through which activation in one domain spreads to another [25]. Applied to the present context, a bipartite network combining cyber victimization items and depressive symptoms allows us to ask: Which specific online harm most strongly activates which specific depressive symptom? Recent applications of bridge analysis in clinical samples have shown that cross‐community bridge nodes are often not the same as intracommunity central nodes, suggesting that the symptom most important for sustaining DEP within its own network may differ from the symptom through which external stressors enter that network [26, 27].

### 1.3. Heterogeneity in Adolescent Risk Profiles

A persistent limitation of network research in adolescent psychopathology is its treatment of samples as homogeneous. Network structure averaged across individuals with very different resource profiles may not represent the architecture of DEP for any particular subgroup. Latent profile analysis (LPA) addresses this directly. Rather than controlling for SE or SM use as covariates, LPA identifies naturally occurring subgroups whose members share a common configuration of these variables [28]. This person‐centered approach preserves the combinatorial logic of risk. Low SE in a context of high SM use may produce a qualitatively different risk pattern than either factor alone. Using a person‐oriented approach, Palermiti et al. [29] identified distinct SE profiles that varied with adolescents’ involvement in cyberbullying experiences. Combining LPA with network comparison tests (NCTs) allows us to examine whether the architecture of DEP, not just its average severity, differs across these subgroups. If adolescents with low SE and high SM use show a denser depressive symptom network than their peers, with stronger intersymptom connections and greater global strength, this would suggest that their DEP is structurally more self‐sustaining and potentially more resistant to single‐symptom interventions. No study has yet integrated LPA‐derived profiles with both NCT and bipartite bridge analyses in the context of cyber victimization and DEP.

### 1.4. The Present Study

The present study pursued four interconnected aims with a community sample of 622 Turkish adolescents. First, we identified latent profiles based on SE, family affluence, and SM use. Second, we estimated the Kutcher Adolescent Depression Scale–6 (KADS‐6) depressive symptom network for the full sample and examined which symptoms were most central. Third, we tested whether network structure and global strength differed across the identified profiles and by gender using permutation‐based NCT. Fourth, we conducted a bipartite bridge network analysis to identify which specific cyber victimization behaviors and depressive symptoms served as the strongest cross‐community connectors.

We expected that adolescents with low SE would show denser, more interconnected DEP networks than higher SE profiles. Such a pattern would indicate a structurally less resilient symptom system. We also expected that high SM use would amplify network density independently of SE. Regarding bridge nodes, we hypothesized that victimization experiences involving public shaming and humiliation, specifically embarrassing messages and photograph sharing, would show the strongest connections to the worthlessness and hopelessness cluster within the DEP network, given the established role of shame‐based appraisals in linking online harassment to depressive cognitions.

## 2. Method

### 2.1. Participants and Procedure

This cross‐sectional study was conducted with 622 adolescents (342 female, 55.0%; 280 male, 45.0%) recruited from secondary schools in urban and suburban regions of Elazig province, Turkey. Schools were not sampled at random: After ethical approval was obtained, secondary schools in the province were invited to take part, and those whose administrations agreed to participate served as the study sites. Within each participating school, all students who attended on the day of administration, whose legal guardians returned written consent, and who themselves gave assent were eligible to participate, and no student was excluded on the basis of psychiatric history, academic standing, or previous experience of online victimization. The sample size was not fixed in advance by a power analysis. All eligible students in the schools that agreed to take part were invited, so the achieved sample size was determined by the number of participating schools and their enrollment. Recruitment and data collection were carried out over a single academic semester (the ___ semester of the 20__–20__ academic year), and each participant completed the survey once. Participants ranged in age from 10 to 19 years (*M* = 13.90 and SD = 1.54). Data were collected via a secure online survey platform administered under the supervision of trained school counselors during regular school hours. It took participants ~15–20 min to complete the scales. Participation was entirely voluntary and anonymous and conducted in accordance with the Declaration of Helsinki. Written informed consent was obtained from both adolescents and their legal guardians prior to data collection. Ethical approval was granted by the Siirt University Institutional Review Board (Protocol Number 1180).

### 2.2. Measures

An overview of all measures, including item count, response format, and internal consistency estimates for the current sample, is provided in Table 1.

**Table 1:** Summary of measures.

Measure	Items	Response scale	*α* (*current sample*)	Reference
Kutcher Adolescent Depression Scale–6 (KADS‐6)	6	0–3 (Likert)	0.806	[30]
Rosenberg Self‐Esteem Scale (RSES)	10	1–4 (Likert)	0.868	[31]
Revised Cyber Bullying Inventory – Victimization (RCBI‐V)	10	1–4 (Likert)	0.794	[32]
Revised Cyber Bullying Inventory – Perpetration (RCBI‐P)	10	1–4 (Likert)	0.822	[32]
Family Affluence Scale II (FAS II)	4	0–9 (composite)	—	[33]
Marlowe–Crowne Social Desirability Scale Short Form C (MCSDS)	13	Binary (Yes/No)	0.601	[34]

*Note: α*, Cronbach’s alpha for the current sample—indicates a composite score for which alpha is not applicable.

Abbreviations: FAS II, Family Affluence Scale II; KADS‐6, Kutcher Adolescent Depression Scale–6 item version; MCSDS, Marlowe–Crowne Social Desirability Scale Short Form C; RCBI, Revised Cyber Bullying Inventory; RSES, Rosenberg Self‐Esteem Scale.

#### 2.2.1. DEP

Depressive symptoms were measured with the Kutcher Adolescent DEP Scale – 6 item version [30]. The KADS‐6 assesses six core depressive symptom clusters: low mood, worthlessness/hopelessness, fatigue, anhedonia, anxiety/tension, and self‐harm/suicidal ideation. Each item is rated on a four‐point scale ranging from 0 (almost never) to 3 (all of the time). A total score of 6 or above is considered indicative of probable DEP. In the original validation study, the six‐item version reached 92% sensitivity and 71% specificity at a cut‐off score of 6 [30]. The Turkish adaptation was carried out by Kaya et al. [35]. Internal consistency in the current sample was *α* = 0.806.

#### 2.2.2. SE

Global SE was assessed using the Rosenberg SE Scale (RSES; [31]), a widely used 10‐item measure evaluating general feelings of self‐worth. Items are rated on a four‐point Likert scale from 1 (strongly disagree) to 4 (strongly agree), with five items reverse‐scored. Higher total scores indicate greater SE (range: 10–40). The Turkish adaptation was conducted by Cuhadaroglu [36]. Internal consistency in the current sample was *α* = 0.868.

#### 2.2.3. Cyber Victimization and Perpetration

Cyber victimization and cyberbullying perpetration were assessed using the Revised Cyber Bullying Inventory (RCBI; [32]). The RCBI includes 10 items measuring the frequency of cyber victimization (RCBI‐V) and 10 parallel items measuring perpetration (RCBI‐P), covering behaviors such as account hacking, threatening messages, embarrassing content sharing, and impersonation. Participants rated the frequency of each behavior over the preceding 6 months on a four‐point scale (1 = never, 2 = once, 3 = twice to three times, and 4 = more than three times). Internal consistency in the current sample was *α* = 0.794 for victimization and *α* = 0.822 for perpetration. For network analysis, only the cyber victimization subscale (RCBI‐V) was used.

#### 2.2.4. Family Affluence

Socioeconomic status was measured using the Family Affluence Scale II (FAS II; [33]), a four‐item self‐report measure assessing material household resources (car ownership, personal bedroom, holiday travel, and computer availability). Composite scores range from 0 to 9, with scores of 0–2 indicating low affluence, 3–5 indicating middle affluence, and 6–9 indicating high affluence, following the classification recommended by Boyce et al. [33].

#### 2.2.5. Social Desirability

Social desirability response bias was assessed using the Marlowe–Crowne Social Desirability Scale Short Form C (MCSDS; [34]), a 13‐item binary (yes/no) measure of the tendency to respond in a socially approved manner. The MCSDS was administered to monitor socially desirable responding, and its associations with the study variables were examined in the preliminary analyses. Internal consistency in the current sample was *α* = 0.593, consistent with previously reported estimates for this brief form.

#### 2.2.6. Additional Variables

Daily SM use was assessed via a single self‐report item, asking participants to estimate the average number of minutes per day they spend on SM platforms (e.g., Instagram, TikTok, and YouTube). Academic performance was assessed using self‐reported grade point average (GPA) on a 0–100 scale, consistent with the Turkish national grading system. Age and gender (1 = female and 2 = male) were recorded as demographic variables and used in the descriptive and group‐comparison analyses.

### 2.3. Data Analysis

#### 2.3.1. Preliminary Analyses

Missing data were assessed using Little’s [37] MCAR test. Given that data were not missing completely at random (*χ* ^2^(224) = 303.54, *p*  < 0.001), missing values were handled using Multiple Imputation by Chained Equations (MICE; [38]), implemented in the mice package (*m* = 20 imputed datasets, maxit = 50 iterations, predictive mean matching method). All subsequent analyses were conducted on pooled estimates across the 20 imputed datasets. Univariate outliers were identified using *z*‐scores (±3.29 criterion) and winsorized to the nearest nonoutlying value. Internal consistency was estimated using Cronbach’s *α* for all scales.

#### 2.3.2. LPA

LPA was conducted using the tidyLPA package [39] to identify latent subgroups of adolescents based on SE (RSES total score), family affluence (FAS II total score), and daily SM use. LPA is a person‐centered statistical method that classifies individuals into homogeneous subgroups (profiles) based on observed variable patterns, without requiring a priori group definitions [28]. Models with one to four profiles were estimated using equal variances and zero covariances (Model 1 in tidyLPA terminology), following best‐practice recommendations for exploratory LPA [28]. Model selection was based on the Bayesian information criterion (BIC; lower values indicate better fit), entropy (values ≥ 0.70 indicate acceptable classification accuracy), the bootstrap likelihood ratio test (BLRT), and the requirement that each profile contain at least 5% of the total sample to ensure adequate statistical power for subsequent analyses. Mean differences in DEP, SE, SM use, and demographic variables across the retained profiles were tested using one‐way ANOVA with Tukey HSD post hoc comparisons and chi‐square tests for gender distribution.

#### 2.3.3. Depressive Symptom Network Estimation

A Gaussian graphical model (GGM) was estimated for the six KADS‐6 depressive symptom items using regularized partial correlations. Network estimation was performed using the estimateNetwork function from the bootnet package [40], with the EBICglasso algorithm (extended BIC graphical LASSO; [41]) for regularization and sparsification. Spearman correlations were used as the input correlation matrix to accommodate the ordinal nature of the items. The EBIC tuning parameter was set to *γ* = 0.5, the recommended default for achieving a balance between model parsimony and edge retention [42]. In the estimated network, nodes represent individual depressive symptoms, and edges represent regularized partial correlations between symptom pairs after controlling for all other symptoms in the network. Edge thickness is proportional to the magnitude of the partial correlation.

Strength and closeness centrality indices were computed using the qgraph package [43] and reported as standardized *z*‐scores to allow comparison across nodes. Strength centrality reflects the sum of absolute edge weights connecting a node to all other nodes, indicating overall connectivity. Closeness centrality reflects how efficiently a node is connected to all others via the shortest paths. Betweenness centrality was computed but not interpreted, as its case‐dropping stability coefficient (CS‐coefficient = 0.127) fell below the acceptable threshold of 0.25 [40]. Network accuracy and stability were evaluated using nonparametric bootstrapping (2500 iterations) to generate 95% confidence intervals for edge weights and case‐dropping subset bootstrapping (2500 iterations) to compute CS‐coefficients for each centrality index. Two sensitivity analyses were carried out for the depressive symptom network. First, the network was re‐estimated with EBIC tuning parameters of *γ* = 0 and *γ* = 0.25 alongside the value of 0.5 used in the main analysis. Second, it was re‐estimated on complete cases only, that is, without multiple imputation. Edge weights, centrality values, and the rank order of the symptoms were compared across these conditions.

#### 2.3.4. NCTs

NCTs [44] were conducted to examine whether the depressive symptom network differed across gender (female vs. male) and across the three latent profiles identified in the LPA, using the NetworkComparisonTest package. The NCT uses permutation‐based resampling to test for statistically significant differences between two networks on two aspects: (a) global network strength invariance, which tests whether the overall magnitude of all edges differs between groups, and (b) network structure invariance, which tests whether the pattern of specific edge differences is greater than would be expected by chance. Each NCT was conducted using 1000 permutations, with a significance threshold of *α* = 0.05.

#### 2.3.5. Bridge Network Analysis

To examine which specific nodes serve as critical cross‐community connectors between the cyber victimization and DEP domains, a bipartite bridge network analysis was conducted. The combined 16‐node network, comprising 10 cyber victimization items (RCBI‐V) and six depressive symptoms (KADS‐6), was estimated using EBICglasso (*γ* = 0.5) with Spearman correlations. Community assignments were defined a priori according to construct membership: nodes 1–10 assigned to the cyber victimization community and nodes 11–16 assigned to the DEP community. Bridge expected influence (1‐step; [45]) was computed using the bridge function from the networktools package. Bridge expected influence (1‐step) is defined as the sum of all edge weights connecting a given node to nodes in other communities (with sign preserved), providing a signed measure of cross‐community influence that is appropriate when negative edges are present in the network. Higher Bridge EI values indicate that a node exerts stronger influence across community boundaries and may therefore represent a clinically meaningful pathway through which stressors in one domain activate symptoms in another.

All analyses were conducted in R (version 4.3.1; [46]) using the following packages: *bootnet* and *qgraph* for network estimation and visualization [40, 43], *networktools* for bridge centrality [25], *NetworkComparisonTest* for permutation‐based group comparisons [44], *tidyLPA* for LPA [39], and *mice* for multiple imputation [38].

## 3. Results

### 3.1. Preliminary Analyses

Descriptive statistics for all study variables are presented in Table 2. Participant flow is summarized in Figure S1. All 622 adolescents who completed the survey were retained in the analytic sample, and no questionnaire was excluded before analysis. The participating schools did not keep records of the number of students who were invited but absent on the day of administration or of the number whose consent forms were not returned, so these figures cannot be reported. The final analytic sample comprised 622 adolescents (55.0% female; *M*_aɡe_ = 13.90, SD = 1.54, range = 10–19). Missing data ranged from 0.5% to 10.5% at the item level and from 0.8% to 21.1% at the scale level. Little’s [37] MCAR test indicated that data were not missing completely at random (*χ* ^2^(224) = 303.54, *p*  < 0.001); therefore, missing values were handled using MICE (*m* = 20, maxit = 50, predictive mean matching). Internal consistency estimates were acceptable to excellent across all measures: DEP/KADS‐6 (*α* = 0.806), SE/RSES (*α* = 0.868), cyber victimization (*α* = 0.794), and cyberbullying (*α* = 0.822). The Marlowe–Crowne Social Desirability Scale demonstrated lower internal consistency (*α* = 0.593), consistent with its binary response format. Cyber victimization and cyberbullying exhibited positive skewness (0.84 and 1.24, respectively), reflecting the low base‐rate nature of cyberbullying in community samples.

**Table 2:** Descriptive statistics for all study variables (*N* = 622).

Variable	*n*	*M*	SD	Min	Max	Skewness	Kurtosis	*α*
1. Depression (KADS‐6)	622	8.74	4.80	0	18	−0.05	−0.73	0.806
2. Self‐esteem (RSES)	622	20.53	6.94	10	40	0.49	−0.45	0.868
3. Cyber victimization	622	16.29	5.96	10	36	0.84	−0.09	0.794
4. Cyber bullying	622	15.60	5.85	10	35	1.24	1.07	0.822
5. Family affluence (FAS II)	622	5.51	1.16	3	9	0.46	−0.31	—
6. Social desirability (MCSDS)	622	6.7	3.22	1	13	−0.27	0.13	0.593
7. GPA	622	88.01	12.40	50	100	−1.25	0.72	—
8. Social media use (min/day)	622	126.77	105.04	0	480	1.36	1.24	—
9. Age	622	13.90	1.54	10	19	0.18	−0.36	—

*Note: α*, Cronbach’s alpha—indicates composite scores for which alpha is not applicable.

Abbreviations: KADS‐6, Kutcher Adolescent Depression Scale–6 item version; FAS II, Family Affluence Scale II; GPA, grade point average; MCSDS, Marlowe–Crowne Social Desirability Scale Short Form C; RCBI, Revised Cyber Bullying Inventory; RSES, Rosenberg Self‐Esteem Scale; SM, social media.

### 3.2. LPA

**Figure 1:**
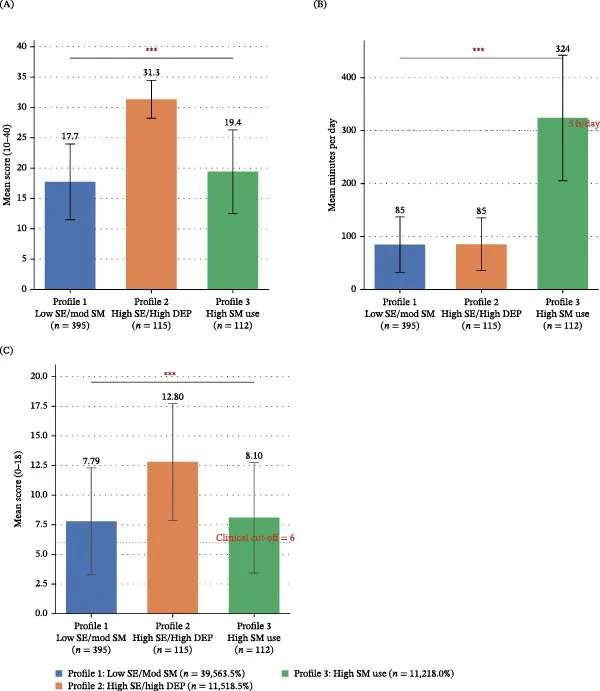
Profile characteristics across the three latent profiles: mean self‐esteem, social media use, and depression scores (*N* = 622). *Note*: DEP, depression; SE, self‐esteem; SM, social media. Error bars represent ±1 standard deviation.  ^∗∗∗^ *p*  < 0.001 (one‐way ANOVA with Tukey HSD post hoc comparisons). Dotted lines indicate the KADS‐6 clinical cut‐off (total score ≥ 6) and a social media threshold of 5 h per day. Family affluence did not differ across profiles (*p* = 0.468) and is therefore not displayed. (A) Self‐esteem (RSES). (B) Daily social media use. (C) Depression (KADS‐6).

LPA was conducted using the tidyLPA package in R to identify distinct adolescent subgroups based on SE, family affluence, and daily SM use. Models with one to four profiles were estimated. Model fit indices are presented in Table 3. Statistical fit continued to improve with each additional profile. The four‐profile model returned the lowest BIC (5163 vs. 5191 for the three‐profile solution) and a significant BLRT (*p*  < 0.001). Its smallest class, however, contained fewer than 5% of the sample (*n* < 31), below the minimum class size specified a priori in the analytic plan. A class this small is unstable across bootstrap replications and would leave the subsequent profile comparisons underpowered. The four‐profile solution was therefore not retained, and the three‐profile model was selected as the most parsimonious admissible solution (BIC = 5191 and entropy = 0.721), with entropy above the conventional 0.70 threshold. Average posterior assignment probabilities for the three retained profiles were 0.887, 0.820, and 0.871, respectively. Profile characteristics are presented in Table 4 and illustrated in Figure 1.

**Table 3:** Latent profile analysis model fit indices (*N* = 622).

Profiles	AIC	BIC	Entropy	BLRT *p*	Smallest class (%)	Selected
1	5304	5331	—	—	100	—
2	5215	5259	0.683	<0.001	34.1	—
3	5129	5191	0.721	<0.001	18.0	**✓**
4	5083	5163	0.742	<0.001	<5	—

*Note:* Fit indices improved monotonically from the one‐ to the four‐profile model. The three‐profile solution was retained as the most parsimonious admissible model: The four‐profile solution produced a lower BIC and a significant BLRT, but one of its classes comprised <5% of the sample (*n* < 31), below the minimum class size specified a priori.

Abbreviations: BIC, Bayesian information criterion; BLRT, bootstrap likelihood ratio test.

**Table 4:** Characteristics of the three latent profiles (*N* = 622).

Variable	Profile 1 low SE/mod SM (*n* = 395, 63.5%)	Profile 2 high SE/high DEP (*n* = 115, 18.5%)	Profile 3 high SM use (*n* = 112, 18.0%)	*F* (df)
Self‐esteem (*M*, SD)	17.73 (6.24)	31.34 (3.12)	19.42 (6.87)	126.4 (2, 619) ^∗∗∗^
Family affluence (*M*, SD)	5.49 (1.21)	5.66 (1.18)	5.55 (1.31)	0.76 (2, 619)
Social media use (min, *M*, SD)	84.72 (52.3)	85.40 (49.8)	323.93 (118.6)	498.2 (2, 619) ^∗∗∗^
Depression (*M*, SD)	7.79 (4.51)	12.80 (4.93)	8.10 (4.67)	57.6 (2, 619) ^∗∗∗^
Female, *n* (%)	196 (49.6%)	66 (57.4%)	79 (70.5%)	*χ* ^2^(2) = 14.8 ^∗∗∗^

*Note*: *F* statistics reflect one‐way ANOVA. Post hoc Tukey HSD: Profile 2 > Profiles 1 and 3 on depression (both *p*  < 0.001).

Abbreviations: DEP, depression; SE, self‐esteem; SM, social media.

^∗∗∗^ *p*  < 0.001.

Profile 1 (low SE/moderate SM; *n* = 395, 63.5%) was characterized by below‐average SE (*M* = 17.73 and SD = 6.24) and moderate SM use (*M* = 84.72 min/day). Profile 2 (high SE/high; *n* = 115, 18.5%) displayed markedly high SE (*M* = 31.34 and SD = 3.12) alongside the highest DEP scores (*M* = 12.80 and SD = 4.93). Profile 3 (high SM use; *n* = 112, 18.0%) was distinguished by substantially elevated daily SM engagement (*M* = 323.93 min/day and SD = 118.6). One‐way ANOVAs confirmed significant between‐profile differences in SE, *F* (2, 619) = 126.4, *p*  < 0.001; SM use, *F* (2, 619) = 498.2, *p*  < 0.001; and DEP, *F* (2, 619) = 57.6, *p*  < 0.001. Post hoc Tukey HSD comparisons indicated that Profile 2 showed significantly higher DEP than both Profile 1 (mean difference = 5.01, *p*  < 0.001, *d* = 1.05) and Profile 3 (mean difference = 4.71, *p*  < 0.001, *d* = 0.99). Profiles 1 and 3 did not differ significantly on DEP (*p* = 0.802). Family affluence did not differ across profiles, *F* (2, 619) = 0.76, *p* = 0.468. Profile characteristics are summarized in Table 4.

### 3.3. Depressive Symptom Network

**Figure 2:**
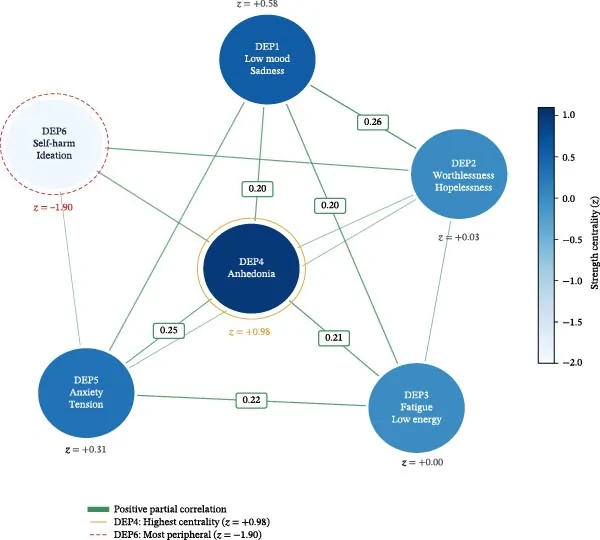
KADS‐6 depressive symptom network estimated using EBICglasso (*N* = 622). *Note*: Network estimated using EBICglasso regularization with Spearman correlations (*γ* = 0.5; *N* = 622). Edge thickness is proportional to the magnitude of the regularized partial correlation; values are shown for the six strongest edges. Node color intensity reflects strength centrality: Darker nodes are more central. Green edges indicate positive partial correlations; no negative edges were retained after regularization (13 of 15 possible edges retained). DEP6 (self‐harm/suicidal ideation) is outlined with a dashed border to highlight its peripheral status within the network (strength *z* = −1.90). DEP1, low mood; DEP2,worthlessness/hopelessness; DEP3, fatigue; DEP4, anhedonia; DEP5, anxiety/tension; DEP6, self‐harm/suicidal ideation.

A GGM was estimated for the six KADS‐6 depressive symptoms using the EBICglasso algorithm (*γ* = 0.5) with Spearman correlations, implemented in the bootnet package. The regularization yielded a sparse network with 13 of 15 possible edges retained (86.7% density). The strongest intersymptom connections were between low mood and worthlessness (partial *r* = 0.257), anhedonia and anxiety (partial *r* = 0.251), anhedonia and fatigue (partial *r* = 0.206), and low mood and fatigue (partial *r* = 0.204). The network is displayed in Figure 2. Centrality indices are presented in Table 5. Anhedonia (DEP4) emerged as the most central symptom (strength *z* = 0.980; closeness *z* = 1.147), indicating that it maintains the strongest direct connections to all other depressive symptoms and is most efficiently connected to the broader network. Low mood (DEP1) was the second most central symptom (strength *z* = 0.579; closeness *z* = 1.102). By contrast, self‐harm/suicidal ideation (DEP6) was the most peripheral symptom (strength *z* = −1.900; closeness *z* = −1.461). Stability analyses confirmed that strength (CS‐coefficient = 0.595) and closeness (CS‐coefficient = 0.439) centrality estimates were reliable. Betweenness was not interpreted due to a stability coefficient below the acceptable threshold (CS = 0.127 < 0.25; [40]). The sensitivity analyses supported these results. Edge weights obtained with *γ* = 0 and *γ* = 0.25 correlated 0.99 with those of the main model, global strength varied only between 2.31 and 2.41, and the strongest edge ranged from 0.253 to 0.256 across the three tuning values. Re‐estimating the network on complete cases (*n* = 591) instead of the imputed data yielded an edge‐weight correlation of 0.98 and a rank correlation of 0.94 for strength centrality. Anhedonia was the most central and self‐harm/suicidal ideation the most peripheral symptom in every condition.

**Table 5:** Network centrality indices for KADS‐6 depressive symptoms (*N* = 622).

Symptom	Strength	*Strength* (*z*)	Closeness	*Closeness* (*z*)
DEP4—anhedonia	0.910	0.980	0.0039	1.147
DEP1—low mood	0.843	0.579	0.0038	1.102
DEP5—anxiety	0.799	0.306	0.0036	−0.200
DEP2—worthlessness	0.726	0.029	0.0034	−0.543
DEP3—fatigue	0.724	0.004	0.0035	−0.045
DEP6—self‐harm ideation	0.419	−1.900	0.0029	−1.461

*Note:* Centrality indices estimated from the EBICglasso network. *z* = standardized centrality values. Betweenness is not reported (CS, coefficient = 0.127 < 0.25). Symptoms ordered by strength centrality. CS, coefficients: strength = 0.595; closeness = 0.439.

### 3.4. NCTs

NCTs [44] were conducted across gender and across the three latent profiles using 1000 permutations. Results are presented in Table 6. Gender comparison revealed no significant differences in global strength (ΔGS = 0.17, *p* = 0.415) or network structure (*p* = 0.404). Profile 1 and Profile 2 differed significantly in global strength (ΔGS = 0.66, *p* = 0.044), with Profile 1 showing a denser network (GS = 2.38) than Profile 2 (GS = 1.72). Profile 1 and Profile 3 demonstrated a significant structural difference (*p* = 0.018), indicating that the configuration of symptom connections differed between these groups. The most pronounced difference was between Profiles 2 and 3 (ΔGS = 0.90, *p* = 0.001).

**Table 6:** Network comparison test results across gender and latent profiles.

Comparison	GS group 1	GS group 2	ΔGS	GS *p*	Structure *p*
Female vs. male	2.28	2.45	0.17	0.415	0.404
Profile 1 vs. Profile 2	2.38	1.72	0.66	0.044 ^∗^	0.146
Profile 1 vs. Profile 3	2.38	2.62	0.24	0.259	0.018 ^∗^
Profile 2 vs. Profile 3	1.72	2.62	0.90	0.001 ^∗∗^	0.086

*Note:* All NCTs used 1000 permutations. Profile 1 = low self‐esteem/moderate social media (*n* = 395); Profile 2 = high self‐esteem/high depression (*n* = 115); Profile 3 = high social media use (*n* = 112). ΔGS, absolute difference in global strength; GS, global network strength.

Abbreviation: NCT, network comparison test.

^∗^ *p*  < 0.05.

^∗∗^ *p*  < 0.01.

### 3.5. Bridge Network Analysis

**Figure 3:**
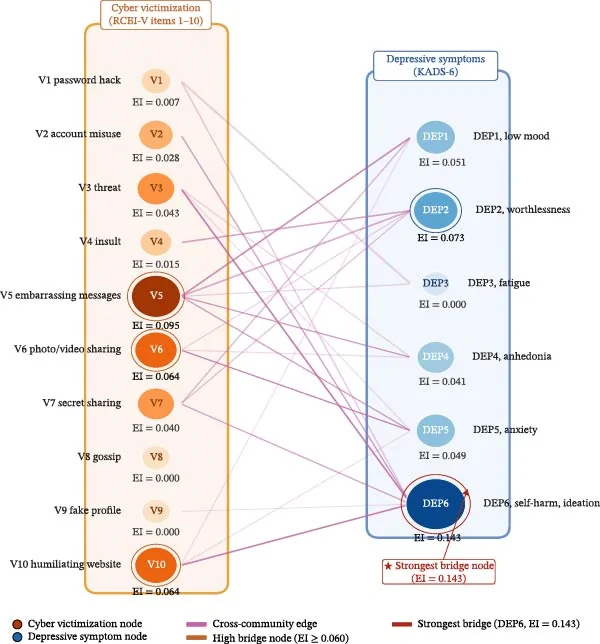
Bipartite bridge network: cyber victimization and KADS‐6 depressive symptoms (*N* = 622). *Note*: Node size and color intensity are proportional to bridge expected influence (1‐step; [45]), shown beneath each node. Orange nodes, cyber victimization community (V1–V10); blue nodes, depression community (DEP1–DEP6). Only cross‐community edges are displayed; edge thickness reflects the magnitude of the regularized partial correlation between the two communities. DEP6 (self‐harm/suicidal ideation) was the strongest cross‐community bridge node (EI = 0.143) despite being the most peripheral node within the depression network, and V5 (embarrassing messages) was the strongest victimization‐side bridge node (EI = 0.095). Gossip (V8), fake profile (V9), and fatigue (DEP3) showed no cross‐community bridge influence (EI = 0.000).

A bipartite bridge network was estimated combining the 10 cyber victimization items (RCBI‐V) and 6 KADS‐6 symptoms (16 nodes total). Bridge expected influence (1‐step) was computed using the networktools package, with community assignments defined a priori. Results are presented in Table 7. Self‐harm/suicidal ideation (DEP6) was the strongest bridge node (EI = 0.143), a striking finding given that this symptom was the most peripheral node within the DEP‐only network. Embarrassing messages (V5; EI = 0.095) was the strongest victimization‐side bridge node, followed by humiliating website (V10) and photo/video sharing (V6; both EI = 0.064). On the DEP side, worthlessness/hopelessness (DEP2; EI = 0.073) was the second strongest bridge symptom. Gossip (V8) and fake profile (V9) showed zero bridge influence (EI = 0.000), indicating no direct cross‐community connections. The full bipartite network is displayed in Figure 3.

**Table 7:** Bridge expected influence values: top 10 bridge nodes (*N* = 622).

Rank	Node	Community	Bridge EI (1‐step)
**1**	**DEP6—self-harm/suicidal ideation**	**Depression**	**0.143**
2	V5—embarrassing messages	Cyber victimization	0.095
3	DEP2—worthlessness/hopelessness	Depression	0.073
4	V10—humiliating website	Cyber victimization	0.064
5	V6—photo/video sharing	Cyber victimization	0.064
6	DEP1—low mood/sadness	Depression	0.051
7	DEP5—anxiety/tension	Depression	0.049
8	V3—threats	Cyber victimization	0.043
9	DEP4—anhedonia	Depression	0.041
10	V7—secret sharing	Cyber victimization	0.040

*Note:* Bridge expected influence (1‐step) computed using the networktools package [45]. Community assignments: cyber victimization (V1–10) and depression (DEP1–DEP6). Bold = highest bridge node. V, cyber victimization item; DEP, depressive symptom.

## 4. Discussion

This study set out to map the pathways from cyber victimization to adolescent DEP using a combination of person‐centered profiling, symptom network analysis, and bipartite bridge analysis in a community sample of Turkish adolescents. Three findings stand out. First, anhedonia emerged as the most central symptom within the depressive symptom network, replicating a pattern observed across multiple adolescent network studies. Second, the depressive symptom network differed significantly in both structure and global strength across the latent profiles. Adolescents in the high SM use group showed the densest intersymptom connections, which aligns with emerging network‐level evidence on digital media and depressive symptomatology. Third, and most theoretically consequential, self‐harm/suicidal ideation was the strongest cross‐community bridge node linking cyber victimization to DEP, despite being the most peripheral symptom within the DEP network itself. This dissociation between intranetwork centrality and cross‐community bridge influence is the central contribution of the present study.

### 4.1. Anhedonia as the Core Depressive Symptom

The finding that anhedonia and low mood were the most central symptoms in the KADS‐6 network replicates and extends prior network analyses of adolescent DEP. Manfro et al. [47] identified sad mood and worthlessness as the most central items in the PHQ‐A among Brazilian adolescents, while Liang et al. [48] found fatigue and depressed mood to be core items in large Chinese adolescent samples. Perrin et al. [49] similarly observed that anhedonia replaced guilt as the second most influential node between 1 and 2 years after traumatic brain injury in veterans and service members, which suggests that anhedonia grows in centrality as depressive states persist over time. The present study adds convergent evidence from a Turkish community adolescent sample. The prominent role of anhedonia in depressive symptom networks may therefore hold across cultural contexts. This cross‐cultural consistency points to a shared structural pattern in adolescent DEP, in which anhedonia appears to help stabilize the depressive state.

From a network theory perspective, high centrality means that anhedonia maintains the strongest connections to all other depressive symptoms, a pattern consistent with its functional role in sustaining depressive states [24]. Schettino et al. [50] provided experimental evidence for this mechanism. Experimentally induced repetitive negative thinking attenuated reward responsiveness and its electrophysiological markers, most strongly in individuals with more severe depressive symptoms. Anhedonia and cognitive rumination appear to form a mutually reinforcing cycle. When anhedonia is present, reward signals are dampened; this, in turn, sustains the negative cognitive states that reinforce low mood and fatigue. Bard et al. [51], examining a comorbid insomnia–DEP–anxiety network, similarly found that low energy and depressive affect symptoms, among them anhedonia, feelings of failure or guilt, and depressed mood, were key predictors of functional impairment across multiple domains, adding to the case that anhedonia carries clinical significance beyond its symptom severity alone.

The clinical implication follows directly. Behavioral activation strategies that increase engagement with rewarding activities and interrupt the ruminative cycles suppressing reward processing may produce the broadest symptom‐level effects because they target the most densely connected node in the network. This recommendation converges with established cognitive–behavioral approaches to adolescent DEP, where activity scheduling is a core component precisely because anhedonia functions as a gateway symptom that, when maintained, sustains the broader depressive episode.

These findings can be situated within broader developmental frameworks of adolescent risk behavior. The dual systems perspective describes adolescence as a period in which sensation seeking rises steeply and peaks in late adolescence, whereas self‐regulatory capacity matures more slowly and continues to develop into the twenties [5]. Elevated sensation seeking is, in turn, associated with more intensive engagement in online environments and with risk‐related media use: A meta‐analysis of SM use and adolescent risk behavior reported small‐to‐medium positive associations between SM use and risky behavior generally (*r* = 0.21) as well as with substance use and risky sexual behavior [52]. This developmental profile is relevant to the present results in two respects. First, adolescents who seek stimulation and novelty online plausibly accumulate greater exposure to precisely the behaviors that emerged here as the strongest victimization‐side bridges, namely, embarrassing messages, humiliating websites, and image sharing, all of which involve public exposure. Second, sensation seeking appears to condition the downstream consequences of victimization rather than merely its likelihood: Yu et al. [53] found that the indirect pathway from cyberbullying victimization to nonsuicidal self‐injury via reduced school engagement was significant only among adolescents high in sensation seeking. Read alongside the present bridge findings, this suggests that the route from online harm to self‐harm–related symptoms may be amplified in high‐sensation–seeking adolescents, and it places the centrality of anhedonia in a developmental context: A blunting of reward responsiveness is particularly consequential during a period otherwise defined by heightened reward sensitivity and reward‐seeking behavior.

### 4.2. Latent Profiles and Network Architecture

The three latent profiles revealed a more complex picture than the initial hypotheses suggested. The vulnerability model traditionally suggests that low SE results in DEP, asserting that individuals with poor SE have higher risks for the development of DEP due to negative and derogatory beliefs about themselves [19, 20, 54]. The high SE/high DEP profile (Profile 2) was unexpected. Adolescents in this group reported the highest SE alongside the highest DEP scores. This pattern challenges the straightforward assumption that SE functions as a protective factor against DEP. One interpretation is that high SE in this context reflects a defensive or contingent self‐regard. Such a presentation may buffer against self‐referential negative cognitions without protecting against hedonic and somatic depressive symptoms. Alternatively, this profile may capture adolescents whose DEP is driven by factors external to the self‐concept, such as anxious perfectionism, high academic pressure, or specific loss experiences. The comparatively low network density observed in Profile 2 may reflect precisely that pattern, namely, a DEP driven by an external source rather than by self‐sustaining symptom feedback, which in a network framework would appear less dense even when total symptom burden is high [24].

The high SM use profile (Profile 3) showed the densest DEP network and a significantly different structural organization from Profile 1. The NCT structure difference between Profiles 1 and 3 indicates that the pattern of which symptoms connect to which differs between these groups. The difference is structural, not simply one of overall density. This finding aligns with Zhan and Wang [55], who identified four distinct network phenotypes in an intensive longitudinal study of young adults linking digital media use to depressive symptoms: a social comparison phenotype characterized by strong connections between SM browsing and negative self‐evaluation and a passive consumption phenotype defined by associations between noninteractive viewing and anhedonic symptoms. Adolescents who spend over 5 h per day on SM likely engage in similar patterns of passive consumption and social comparison, which create distinctive intersymptom reinforcement cycles. This evidence supports the broader argument that digital media use shapes the architecture of depressive symptoms and not only their severity [55].

### 4.3. The Bridge Network Paradox: Peripheral Yet Critical

The most theoretically important finding is the dissociation between intranetwork centrality and bridge centrality for self‐harm/suicidal ideation (DEP6). Within the KADS‐6 DEP network, DEP6 was the most peripheral node. It had the weakest connections to other depressive symptoms, the lowest strength centrality, and the lowest closeness. By conventional network logic, this would suggest that it is the least important target for intervention. Yet, DEP6 showed the strongest bridge expected influence in the bipartite network, meaning it was the symptom most strongly activated by cyber victimization behaviors across the community boundary. Strathdee et al. [26] reported a structurally analogous pattern in a DEP, anxiety, and fatigue network in multiple sclerosis: at 6‐month follow‐up, anhedonia carried the highest expected influence within the network, whereas the bridging symptoms were restlessness, uncontrollable worry, and suicidal ideation. The symptoms that connected the communities were, therefore, not the ones that dominated within them. Farkas et al. [27] similarly found, in a Hungarian adolescent sample, that cyberbullying victimization was linked to nonsuicidal self‐injury primarily through anxiety rather than through DEP, suggesting that cyber victimization activates specific symptom nodes rather than elevating general psychopathological distress uniformly.

This pattern carries a direct clinical implication. Suicidal ideation does not appear to arise primarily as a downstream consequence of other depressive symptoms cascading through the KADS‐6 network in cyberbullied adolescents. Instead, it appears to be linked more directly to specific online experiences. This is consistent with Cao et al. [56], who found in a sample of 17,633 Chinese students that cyberbullying victimization was associated with suicidal ideation in high school students of both sexes and that hopelessness mediated a substantial share of the victimization–suicidal ideation association, reaching 29.4% for combined family, school, and cyberbullying victimization among male university students. One plausible mechanism involves acute threat to social identity and perceived burdensomeness. Such experiences may bypass the standard depressive pathway and be associated with suicidal cognitions in vulnerable adolescents. Clinicians assessing cyberbullied adolescents should therefore screen explicitly for suicidal ideation even in the absence of prominent depressive symptoms, since standard intranetwork logic would not flag this symptom as a clinical priority.

### 4.4. Embarrassing Messages as the Key Victimization Bridge

On the victimization side, the finding that embarrassing messages showed the strongest bridge influence aligns with theoretical accounts of online harassment and shame‐based appraisals. Unlike account hacking or gossip, which involve violations of information access, embarrassing messages combine public exposure with direct identity threat in a way that may activate cognitive distortions around worthlessness and hopelessness. This is consistent with the cohigh bridge influence observed for worthlessness/hopelessness on the DEP side. The two nodes with the strongest cross‐community influence index the same psychological process of social identity damage. Image‐based and video victimization similarly involves public exposure of private information. This supports the interpretation that the public humiliation dimension of online harm, rather than its frequency or platform, constitutes the primary pathway into the depressive symptom network. Klocek et al. [17] similarly reported form‐specific outcomes, with cyber victimization linked to social phobia and low SE and verbal victimization linked to feelings of worthlessness, which supports the broader point that different victimization forms carry different psychological signatures.

This specificity has practical implications for prevention. Rather than uniform anticyberbullying programs, targeted interventions addressing the sending and sharing of embarrassing content may produce the greatest reduction in victimization‐driven DEP and suicidal ideation. Such interventions include school‐based education on the psychological consequences of digital humiliation, platform reporting mechanisms optimized for this behavior type, and bystander intervention training. The near‐zero bridge influence of gossip and fake profile creation further suggests that not all cyber victimization forms are equally harmful from a depressive symptom activation perspective, which has resource allocation implications for school counseling and public health programming.

### 4.5. Absence of Gender Differences in Network Structure

The NCT found no significant differences in global strength or network structure between female and male adolescents. This null result contrasts with general assumptions about gender differences in adolescent DEP and with findings from some network studies. Zhan and Wang [55] reported that females showed stronger SM–rumination associations than males in their digital media–DEP network, and Panteli et al. [7] found that cyberbullying at age 15 was specifically associated with increased stress among females but not males. In a large US high school sample, associations between bullying victimization and suicidality likewise differed by sex and sexual orientation [57]. The absence of gender differences in the present study may reflect several factors. First, the KADS‐6 is a brief measure focused on core symptom clusters; finer‐grained instruments capturing additional gender‐specific phenomenology, such as rumination, interpersonal sensitivity, or physical symptoms, might reveal structural differences that the KADS‐6 cannot detect. Second, the profile‐level heterogeneity already captured much of the variance associated with SM use, which is itself correlated with gender in this sample (Profile 3 was 70.5% female). Third, gender differences in network structure may be more pronounced in clinical than in community samples or may emerge more clearly in longitudinal designs that track symptom networks across the full adolescent developmental period.

### 4.6. Limitations and Future Directions

Several limitations qualify these findings. The cross‐sectional design precludes causal inference: The bridge connections identified here are statistical associations that cannot confirm the temporal sequence of victimization and symptom activation without longitudinal measurement. The Turkish school–based sample limits generalizability to other cultural contexts where the social meaning of online harm may differ, and the brief KADS‐6 captures only six depressive symptom clusters. Profile enumeration is a further limitation. Both the BIC and the BLRT favored a four‐profile solution, which was set aside because its smallest class fell below the 5% minimum required for the subsequent network comparisons. The three‐profile partition reported here is therefore the most stable solution rather than the statistically optimal one, and a larger sample may support finer differentiation. Future research should replicate this design longitudinally, incorporate clinician‐rated measures alongside self‐report, and examine whether the bridge nodes identified here prospectively predict the onset of clinical DEP following acute victimization episodes.

## 5. Conclusion

Taken together, these findings reframe how the cyber victimization–DEP relationship should be understood. The pathway from online harm to DEP is not uniform: It varies by adolescent profile, is structured differently for those with high SM use, and operates through specific bridge nodes that would not be identified by conventional regression or latent variable approaches. Three clinical messages follow directly. In cyberbullied adolescents, suicidal ideation should be explicitly screened for even when other depressive symptoms are not prominent. Target anhedonia is the symptom most likely to sustain the depressive episode once it is activated. And prioritize prevention efforts against embarrassing and image‐based victimization as the specific online behaviors with the strongest documented pathways into the depressive symptom network.

## Author Contributions

Ergün Kara collected data, conducted analysis, wrote the discussion and literature review parts of the study, and checked the whole research process. Halil Aslan planned the research, conducted analysis, prepared tables and figure, wrote literature review and discussion, and checked the whole research process.

## Funding

This research received no external funding.

## Ethics Statement

The study was conducted in accordance with the Declaration of Helsinki and approved by the Institutional Review Board (or Ethics Committee) of Siirt University on 22.05.2025 (Protocol Number 1180).

## Consent

Informed consent was obtained from all participants and their parents who took part in the study.

## Conflicts of Interest

The authors declare no conflicts of interest.

## Supporting Information

Additional supporting information can be found online in the Supporting Information section.

## Supporting information


**Supporting Information** The high‐resolution figures and supporting files associated with this study have been submitted separately as part of the manuscript files and are available in the online version of this article, including completed STROBE checklist for cross‐sectional studies. Completed the online survey during regular school hours *n* = 670.

## Data Availability

The data that support the findings of this study are available upon request from the corresponding author. The data are not publicly available due to restrictions, for example, their containing information that could compromise the privacy of research participants.
