# Supplementary material for: Pathways From Cyber Victimization to Adolescent Depression: A Latent Profile and Bridge Network Analysis
Source: Depress Anxiety. 2026 Sep 26;2026:3544219. doi: 10.1155/da/3544219 (PMC13615450; doi:10.1155/da/3544219)
Supplement: Supplementary file 1 [file DA-2026-3544219-s001.zip › Sekil_S1_work flow.docx]

**Supplementary Figure S1**

*Participant flow through the study (STROBE item 13)*

| **Secondary schools in Elazığ province invited to take part**  **k = 12** |  |
| --- | --- |
| **↓** |  |
| **Schools whose administrations agreed to participate (study sites)**  **k = 8** | Schools declining or not responding  k =4 |
| **↓** |  |
| **Students enrolled in participating schools and invited to take part**  **n = 750** | Absent on the day of administration  n = 45 |
| **↓** |  |
| **Parental consent returned and student assent given**  **n = 675** | Consent not returned or assent declined  n = 30 |
| **↓** |  |
| **Completed the online survey during regular school hours**  **n = 640** | Did not start or did not complete the survey  n = 5 |
| **↓** |  |
| **Questionnaires excluded before analysis**  **n = 18**  Reason(s): Inattentive responding and duplicate entries |  |
| **↓** |  |
| **Final analytic sample**  **n = 622**  341 girls (54.8%), 281 boys (45.2%)  M age = 13.89, SD = 1.11, range 11–18 |  |
| **↓** |  |
| Missing values 0.3%–2.1% per variable, handled with multiple imputation (MICE, m = 20); no case deleted for missing data |  |
