# Supplementary material for: Pathways From Cyber Victimization to Adolescent Depression: A Latent Profile and Bridge Network Analysis
Source: Depress Anxiety. 2026 Sep 26;2026:3544219. doi: 10.1155/da/3544219 (PMC13615450; doi:10.1155/da/3544219)
Supplement: Supplementary file 1 [file DA-2026-3544219-s001.zip › STROBE_kontrol_listesi.docx]

**STROBE Statement — Checklist of items that should be included in reports of cross-sectional studies**

*Manuscript: Pathways from Cyber Victimization to Adolescent Depression: A Latent Profile and Bridge Network Analysis (ID 3544219)*

*Journal: Depression and Anxiety. Line numbers refer to the continuously numbered revised manuscript. Items in red still require information that only the authors can supply.*

| **Item No.** | **Recommendation** | **Reported in manuscript** | **Notes** |
| --- | --- | --- | --- |
| **Title and abstract** | | | |
| 1 | (a) Indicate the study’s design with a commonly used term in the title or the abstract | Abstract, line 15 | The abstract describes “a cross-sectional community sample of 622 Turkish adolescents”. |
|  | (b) Provide in the abstract an informative and balanced summary of what was done and what was found | Abstract, lines 12–28 | Design, sample, analyses (LPA, NCT, bipartite bridge network) and main findings; within the 300-word limit. |
| **Introduction** | | | |
| 2 | Background/rationale: explain the scientific background and rationale for the investigation being reported | Lines 33–139 | Clinical and epidemiological background, developmental context, cyber victimization and depression, self-esteem and social media use, network and bridge rationale. |
| 3 | Objectives: state specific objectives, including any prespecified hypotheses | Lines 140–155 | Four aims followed by the hypotheses. |
| **Methods** | | | |
| 4 | Study design: present key elements of study design early in the paper | Abstract line 15; Method line 158 | The Participants and Procedure section opens with “This cross-sectional study was conducted with 622 adolescents…”. |
| 5 | Setting: describe the setting, locations, and relevant dates, including periods of recruitment, exposure, follow-up, and data collection | Lines 158–176 | Setting, school selection and study period are reported. ACTION NEEDED: at line 169 replace the bracketed placeholder with the semester and academic year of data collection. |
| 6 | (a) Eligibility criteria, and the sources and methods of selection of participants | Lines 160–165 | Schools were not sampled at random; the schools whose administrations agreed to take part served as study sites. Eligibility: attendance on the day of administration, returned parental consent, and student assent, with no exclusion on the basis of psychiatric history, academic standing or previous online victimization. |
| 7 | Variables: clearly define all outcomes, exposures, predictors, potential confounders, and effect modifiers | Lines 178–232; Table 1 (line 181) | Depression (KADS-6, cut-off ≥ 6, line 187), self-esteem (RSES, line 196), cyber victimization (RCBI-V, line 203), family affluence (FAS II, line 213), social desirability (MCSDS, line 219), social media use, GPA, age and gender (line 226). |
| 8 | Data sources/measurement: for each variable of interest, give sources of data and details of methods of assessment | Lines 158–176; lines 178–232; Table 1 | All measures were self-report, administered on one secure online platform under counsellor supervision, identically for all participants; completion took approximately 15–20 minutes. |
| 9 | Bias: describe any efforts to address potential sources of bias | Lines 158–176; lines 219–225; lines 234–241 | Anonymous and voluntary administration under supervision, social desirability measured with the MCSDS, and screening of missing data and univariate outliers. |
| 10 | Study size: explain how the study size was arrived at | Lines 165–168 | No a priori power analysis was carried out; all eligible students in the schools that agreed to take part were invited, so the achieved sample size was set by the number of participating schools and their enrolment. |
| 11 | Quantitative variables: explain how quantitative variables were handled in the analyses | Lines 234–241; lines 243–256; line 213 | z-score screening with winsorizing at ±3.29; continuous indicators in the LPA; FAS II affluence bands; ordinal items analysed with Spearman correlations. |
| 12 | (a) Describe all statistical methods, including those used to control for confounding | Lines 234–311 | LPA (line 243), GGM/EBICglasso estimation (line 258), centrality and stability (line 269), NCT (line 284), bipartite bridge analysis (line 293), software and packages (line 306). Estimates are unadjusted; no confounder-adjusted models were fitted. |
|  | (b) Describe any methods used to examine subgroups and interactions | Lines 284–291 | Permutation-based Network Comparison Tests across gender and across the three latent profiles (1,000 permutations). |
|  | (c) Explain how missing data were addressed | Lines 234–241; lines 320–328 | Little’s MCAR test followed by multiple imputation by chained equations (m = 20, maxit = 50, predictive mean matching); analyses pooled across imputed datasets. |
|  | (d) If applicable, describe analytical methods taking account of sampling strategy | Not applicable | Convenience school-based sample without survey weighting; school-level clustering was not modelled. |
|  | (e) Describe any sensitivity analyses | Method lines 269–282; Results lines 384–411 | The depressive symptom network was re-estimated with EBIC tuning parameters of γ = 0 and γ = 0.25 and on complete cases without imputation. Edge weights correlated .99 and .98 with the main model, global strength varied between 2.31 and 2.41, and anhedonia remained the most central and self-harm/suicidal ideation the most peripheral symptom in every condition. |
| **Results** | | | |
| 13 | (a) Report numbers of individuals at each stage of the study | Lines 314–318; Supplementary Figure S1 | All 622 adolescents who completed the survey were retained; no questionnaire was excluded. The participating schools did not keep records of the numbers invited, absent, or without returned consent, and this is stated explicitly in the text and in Figure S1. |
|  | (b) Give reasons for non-participation at each stage | Lines 314–318 | The text states that the participating schools did not keep records of the students who were absent or whose consent forms were not returned, so reasons for non-participation cannot be quantified. |
|  | (c) Consider use of a flow diagram | Supplementary Figure S1 | STROBE-style flow diagram supplied as an editable Word file; cited at line 314 and listed under Supplementary Material at line 682. |
| 14 | (a) Give characteristics of study participants and information on exposures and potential confounders | Lines 319–328; Table 2 (line 331); Table 4 (line 370) | Age, gender, GPA, family affluence, social media use, social desirability and all study variables. |
|  | (b) Indicate number of participants with missing data for each variable of interest | Line 320 | Missing data ranged from 0.3% to 2.1% across variables; per-variable figures are not tabulated. |
| 15 | Report numbers of outcome events or summary measures | Lines 319–328; Table 2 | Means, standard deviations, ranges, skewness, kurtosis and internal consistency for all measures. |
| 16 | (a) Give unadjusted estimates and, if applicable, confounder-adjusted estimates and their precision | Lines 356–367 (ANOVA, F, p, Cohen’s d); lines 384–411 and Table 5; lines 419–425 and Table 6; lines 434–443 and Table 7 | All estimates are unadjusted; precision is reported through bootstrapped 95% confidence intervals (2,500 iterations) and case-dropping stability coefficients. |
|  | (b) Report category boundaries when continuous variables were categorized | Line 213; lines 337–367 | FAS II affluence bands; latent profiles derived empirically rather than by cut-off. |
|  | (c) If relevant, consider translating estimates of relative risk into absolute risk | Not applicable | No relative-risk estimates were computed. |
| 17 | Report other analyses done, e.g. analyses of subgroups and interactions, and sensitivity analyses | Lines 419–425 | Gender and profile comparisons, together with the two sensitivity analyses reported under item 12(e). |
| **Discussion** | | | |
| 18 | Key results: summarise key results with reference to study objectives | Lines 477–489 |  |
| 19 | Limitations: discuss limitations of the study, taking into account sources of potential bias or imprecision | Lines 642–654 | Cross-sectional design, single cultural context, brief six-item depression measure. |
| 20 | Interpretation: give a cautious overall interpretation of results considering objectives, limitations, multiplicity of analyses, results from similar studies, and other relevant evidence | Lines 477–654 | Findings are interpreted against comparable network studies; causal language is avoided in line with the cross-sectional design. |
| 21 | Generalisability: discuss the generalisability (external validity) of the study results | Lines 645–654 | Generalisability limited to Turkish school-based community samples. |
| **Other information** | | | |
| 22 | Funding: give the source of funding and the role of the funders for the present study | Line 670 | “This research received no external funding.” Data availability line 671, ethics approval lines 675–679, competing interests line 680, supplementary material lines 681–682. |

*Reference: von Elm E, Altman DG, Egger M, Pocock SJ, Gøtzsche PC, Vandenbroucke JP; STROBE Initiative. Checklist for cross-sectional studies, https://www.strobe-statement.org/checklists/*
